# 

**Published:** 1846-07

**Authors:** 


### BRITISH AND FOREIGN MEDICAL REVIEW

OR

### QUARTERLY JOURNAL

OF

### PRACTICAL MEDICINE AND SURGERY

STATE OF LOUISIANA CHARITY HOSPITAL
JUSTICE
MEDICAL LIBRARY

LIBRARY OF THE
Orleans Parish Medical Society

EDITED BY

JOHN FORBES M.D. F.R.S. F.G.S.

VOL. XXII.

JULY—OCTOBER 1846

LONDON
JOHN CHURCHILL PRINCES STREET SOHO
MDCCCXLVI.

C. AND J. ADLARD, PRINTERS, BARTHOLOMEW CLOSE.

### BOOKS RECEIVED FOR REVIEW.

1. Memoranda on Different Subjects in Anatomy, Surgery, and Pathology. By M. N. Bowen, Surgeon. Second Edition. London, 1846. 24mo, pp. 259. 3s. 6d.

2. A Practical Manual, containing a Description of the general Chemical and Microscopical Characters of the Blood, or Secretions of the Human Body. Part II. By J. W. Griffith, M.D., F.L.S. London, 1846. 8vo, pp. 164. 5s.

3. Remarks on the Dysentery and Hepatitis of India. By E. A. Parkes, M.B. London, 1846. 8vo, pp. 271.

4. Remarks upon Medical Organization and Reform, Foreign and English. By E. Lee. London, 1846. 8vo, pp. 121. 4s. 6d.

5. The Mineral Waters of Kreutznach. By J. E. P. Prieger, M.D. Lond. 1846. 8vo, pp. 92. 3s.

6. Notes and Recollections of a Professional Life. By the late W. Fergusson, M.D. Edited by his son, James Fergusson. London, 1846. 8vo, pp. 248.

7. Flora Capensis. Contributions to the Botany and Topography of Gibraltar. By E. F. Kelaart, F.L.S., F.G.S. Lond. 1846. 8vo, pp. 219.

8. Moral Philosophy; or, the Duties of Man considered in his Individual, Domestic, and Social Qualities. By George Combe. Third Edition. Edinburgh, 1846. Royal 8vo, pp. 116. Double columns, 2s.

9. Outlines of the Course of Qualitative Analysis followed in the Giessen Laboratory. By H. Will. London, 1846. 8vo, pp. 103.

10. Bericht über die durch den Gebrauch des Microscopes in dem Studium der Anatomie und Physiologie erhaltenen Resultate, den Ursprung und die Verrichtungen der Zellen. Von Jakob Paget und Dr. W. B. Carpenter. Aus dem Englischen übersetzt von Dr. Raimond Melzer. Augsburg. 1845. 8vo, pp. 160.

11. Bericht über die Fortschritte der Menschlichen Anatomie und Physiologie im den Jahren 1843-4. Von Jakob Paget. Aus dem Englischen von Dr. Raimond Melzer. Augsburg, 1846. 8vo, pp. 174.

12. Commentary on the Hindu System of Medicine. By T. A. Wise, M.D. Calcutta, 1845. 8vo, pp. 431.

13. Clinical Illustrations of the Diseases of India, as exhibited in the Medical History of a Body of European Troops, for a series of years from their arrival in that country. By William Geddes, M.D. London, 1846. 8vo, pp. 492.

14. Elements of the Theory and Practice of Medicine, designed for the use of Students and Junior Practitioners. By George Gregory, M.D. Sixth Edition. London, 1846. 8vo, pp. 799. 16s.

15. Phrenology considered in a Religious Light. By Mr. John Pugh. London, 1846. 8vo, pp. 208.

